# Bayesian tip-dated timeline for diversification and major biogeographic events in Muroidea (Rodentia), the largest mammalian radiation

**DOI:** 10.1186/s12915-024-02053-2

**Published:** 2024-11-25

**Authors:** Raquel López-Antoñanzas, Tiago R. Simões, Fabien L. Condamine, Moritz Dirnberger, Pablo Peláez-Campomanes

**Affiliations:** 1 Institut Des Sciences de L’Évolution de Montpellier (CNRS/UM/IRD/EPHE), Université de Montpellier, 34095 Montpellier, France; 2 Departamento de Paleobiología, Museo Nacional de Ciencias Naturales-CSIC, Madrid, Spain; 3 Department of Ecology and Evolutionary Biology, Princeton University, Princeton, NJ 08544 USA

**Keywords:** Bayesian tip dating, Divergence dates, Historical palaeobiogeography, Morphological clock, Muroidea, Rodentia

## Abstract

**Background:**

Extinct organisms provide vital information about the time of origination and biogeography of extant groups. The development of phylogenetic methods to study evolutionary processes through time has revolutionized the field of evolutionary biology and led to an unprecedented expansion of our knowledge of the tree of life. Recent developments applying Bayesian approaches, using fossil taxa as tips to be included alongside their living relatives, have revitalized the use of morphological data in evolutionary tree inferences. Eumuroida rodents represent the largest group of mammals including more than a quarter of all extant mammals and have a rich fossil record spanning the last ~ 45 million years. Despite this wealth of data, our current understanding of the classification, major biogeographic patterns, and divergence times for this group comes from molecular phylogenies that use fossils only as a source of node calibrations. However, node calibrations impose several constraints on tree topology and must necessarily make a priori assumptions about the placement of fossil taxa without testing their placement in the tree.

**Results:**

We present the first morphological dataset with extensive fossil sampling for Muroidea. By applying Bayesian morphological clocks with tip dating and process-based biogeographic models, we provide a novel hypothesis for muroid relationships and revised divergence times for the clade that incorporates uncertainty in the placement of all fossil species. Even under strong violation of the clock model, we found strong congruence between results for divergence times, providing a robust timeline for muroid diversification. This new timeline was used for biogeographic analyses, which revealed a dynamic scenario mostly explained by dispersal events between and within the Palearctic and North African regions.

**Conclusions:**

Our results provide important insights into the evolution of Muroidea rodents and clarify the evolutionary pathways of their main lineages. We exploited the advantage of tip dating Bayesian approaches in morphology-based datasets and provided a classification of the largest superfamily of mammals resulting from robust phylogenetic inference, inferring the biogeographical history, diversification, and divergence times of its major lineages.

**Supplementary Information:**

The online version contains supplementary material available at 10.1186/s12915-024-02053-2.

## Background

Establishing an evolutionary timescale is fundamental for tackling a great variety of topics in evolutionary biology, including the reconstruction of patterns of historical biogeography [1], coevolution, and diversification [2]. Without knowledge on the timing of evolutionary events, it is not possible to test hypotheses of causality that invoke extrinsic environmental mechanisms, such as the role of tectonics or climate change in the evolution of the clades [3]. Similarly, it is not possible to determine the rate of evolutionary change in response to such causal factors [4]. The tree of life is pruned by extinction and molecular data cannot be gathered for the vast majority of extinct lineages. Hence, the fossil record once provided the gold standard in attempts to establish evolutionary timescales, which was subsequently complemented by molecular clock approaches for groups with extant representatives [5, 6].

Besides providing fundamental calibration points for critical nodes in divergence time estimates using molecular clocks [3, 7], fossil taxa can now be analyzed either alone—i.e., morphological clocks [8]—or with molecular data in total evidence dating approaches [9, 10]. These advances over the past decade have made it possible to infer the placement of fossil tips without prior assumption of their location in the tree nor employing a priori constraints on tree topology, besides providing more accurate and precise estimate for lineage divergence times across nearly all branches of the tree of life—e.g., [11–16].

Despite the outstanding diversity of rodents among mammals, the richness of their fossil record and their importance to time scaling the molecular trees of living mammals, few attempts have been made to build phylogenies based on morphological data including both extinct and extant rodents [17–19]. Further, no study to date has provided a precise timeline for the evolution of its most speciose clade, Muroidea, which with more than 1695 species (Burgin et al., 2018), is the most diverse group of mammals representing more than a quarter of its total diversity. This group of rodents has a nearly continuous fossil record since the Eocene, which is extremely valuable for informing branch length and, thus, divergence time analyses. These rodents are ubiquitous being distributed in all the geography except for Antarctica. The study of phylogenic relationships inside the muroids is one of the most complex problems that mammalogists have to tackle. Increasing interest in this clade has led to some molecular phylogenetic analyses of living Muroidea using mitochondrial and nuclear genes, which have provided some insights into their evolutionary relationships [20–23]. So, huge efforts on molecular studies made over the past two decades, particularly those including broad taxa sampling [22–25], laid the foundation of a subdivision of muroid rodents in twenty subfamilies included in the following six primary monophyletic groups: Platacanthomyidae, Spalacidae, Calomyscidae, Nesomyidae, Cricetidae, and Muridae [23, 24]. According to recent molecular studies, Platacanthomyidae with only two genera and five living species is the most basal muroid clade. It diverges from the remaining muroid families in the middle Eocene (around 40–45 Ma) [23] whereas its most ancient fossil, the sole extinct genus, †*Neocometes* have been recorded from the early Miocene of South Asia and Europe [26, 27]. Spalacidae or mole rats include three extant subfamilies: the Asian Myospalacinae, the African and Asian Rhizomyinae (tribes Tachyoryctini and Rhizomyini), and the Spalacinae, which live in Eastern Europe, Western Asia, and the Middle East. They represent the second earliest diverging clade inside Muroidea, from which they split during the Oligocene [23]. These results are in line with paleontological data, according to which the oldest known crown spalacid, †*Prokanisamys kowalskii* (Rhizomyinae), would come from the Oligocene of Pakistan [28]. However, the first fossil recognized as a true spalacine, †*Pliospalax macovei*, comes from the Pliocene of Romania [29]. Calomyiscidae, a poorly known monogeneric family, is distributed in southwestern Asia. Due to plesiomorphic morphological dental characters, this group of rodents has been considered by paleontologists as belonging to a stem group inside Gerbillinae, †Myocricetodontinae [30]. However, molecular results point out to a more basal position inside Muroidea, being the sister group of Nesomyidae-Muridae-Cricetidae, from which it diverges circa 20 Ma [23]. Its earliest representatives have been recorded from the Late Miocene of Europe and Turkey [31]. Nesomyidae is divided into six subfamilies Nesomyinae, Delanymyinae, Mystromyinae, Petromyscinae, Cricetomyinae, and Dendromurinae, all but the Magalasy Nesomyinae live in sub-Saharan Africa and Zanzibar island. Due to their morphological diversity and dental convergence, different species inside this group were classified as belonging to Muridae or Cricetidae [32]. However, molecular genetic work has evidenced the monophyly of this group [23, 33], which is the sister clade of the most derived muroids (Muridae plus Cricetidae), from which it diverges circa 19 Ma. The oldest known record of nesomyids represented by the extinct species †*Afrocricetodon songhori* and †*Notocricetodon petteri* [34], comes from the Early Miocene of Africa (Kenya, Uganda, and Namibia). These taxa have been placed inside the extinct subfamily †Afrocricetodontinae, which has been considered to be part of Nesomyidae.

The Muridae, Old World mice and rats, with more than 800 species [35] is the largest family of mammals. Major taxonomic issues concerning the classification of murid rodents resulted from the morphological dental disparity displayed by the teeth of their representatives, so their systematic has been largely discussed for a long time [32, 36–40]. Phylogenetic analysis using molecular data recovered Muridae as distributed in four monophyletic groups, the subfamilies Lophiomyinae, Deomyinae, Gerbillinae, and Murinae, which are generally accepted [22–24]. According to these studies, the most basal murid subfamily is Lophiomyinae followed by Murinae, which is a sister group of Deomyinae plus Gerbillinae. The estimation for the dates of the origin, diversification, and divergence time among and within these subfamilies is still controversial, and molecular results disagree sometimes with paleontological evidences.

The second-most speciose muroid family, with more than 700 living species, corresponds to that of Cricetidae, which brings together New World rats and mice (Sigmodontinae, Neotominae, and Tylomyinae) and Old World hamsters and voles (Cricetinae, Arvicolinae). Molecular studies agree in recovering the sister group relationship of Cricetidae and Muridae but there are still differences in the estimated ages, in which they would have split (e.g., Early Miocene, [23]) versus Late Oligocene [24]. According to molecular results, the diversification of cricetid rodents took place in Late Oligocene times and predates that of murids [24], whereas other authors pointed out that the diversification of cricetids started after that of murids approximately 15 Ma [23]. The issue of estimating divergence ages with accuracy worsens by the inclusion of fossil forms, as there is no consensus regarding the definition of which fossil forms should be included in the family Cricetidae. In fact, it has been a common practice among paleontologists to include inside Cricetidae all fossil forms having a “cricetid” dental pattern and, therefore, to integrate in this group species recorded from the middle Eocene on. However, other researchers consider cricetids exclusively as the most derived forms recorded from the lower Miocene onwards, which probably are more directly related to present-day representatives of this family, an opinion that we share.

All in all, molecular studies dealing on rodents in general and on Muroidea in particular have used fossil taxa merely to indirectly calibrate the divergences (node-dating) between living lineages instead of using the rich fossil record of the group for tip-dated evidence time estimates. Node calibrations are based on the oldest fossil record of a clade, and therefore, they require a prior phylogenetic hypothesis that usually does not exist. Therefore, several different ages have been estimated for the origin and divergence among and within this important group of rodents (e.g., [21, 22, 24, 41]) but no consensus has been reached. Moreover, we still ignore the answer to many important questions concerning the evolutionary origins of the muroid. In fact, we remain ignorant of the phylogenetic position of the fossil taxa with respect to their present-day relatives, which directly impact any node-based fossil calibrations using only molecular data with many deep nodes having poor support. The main reason not to use the fossil species to be included alongside their living relatives in a tip calibration is the lack of morphological data. In fact, the huge number of muroid taxa, their high molar diversity, and morphological convergence of their dental characters have prevented paleontologists and neontologists to build comprehensive phylogenies based on discrete morphological traits including both extinct and extant representatives. It has to be taken in mind that the classical definition of the relationships among fossil rodents is mainly based on dental characters because the teeth are nearly the sole remains preserved in ancient rodents.

Here, we provide the first detailed timeline of muroid evolution and revision of the placement of several of its fossil members by using tip-dated relaxed clock Bayesian inference. Moreover, we use this new timetree to infer their biogeographical history and elucidate the origin and speciation events of the major branches of the muroid tree of life by applying a maximum likelihood inference of geographic range evolution taking into account non-ultrametric dated trees.

## Results and discussion

The analyses we present below include the most extensive paleontological database of muroid rodents and constitute the first attempt to build a phylogeny of Muroidea that includes fossils and extant as tips.

### Phylogenetics: relationships among major clades

**Fig. 1:**
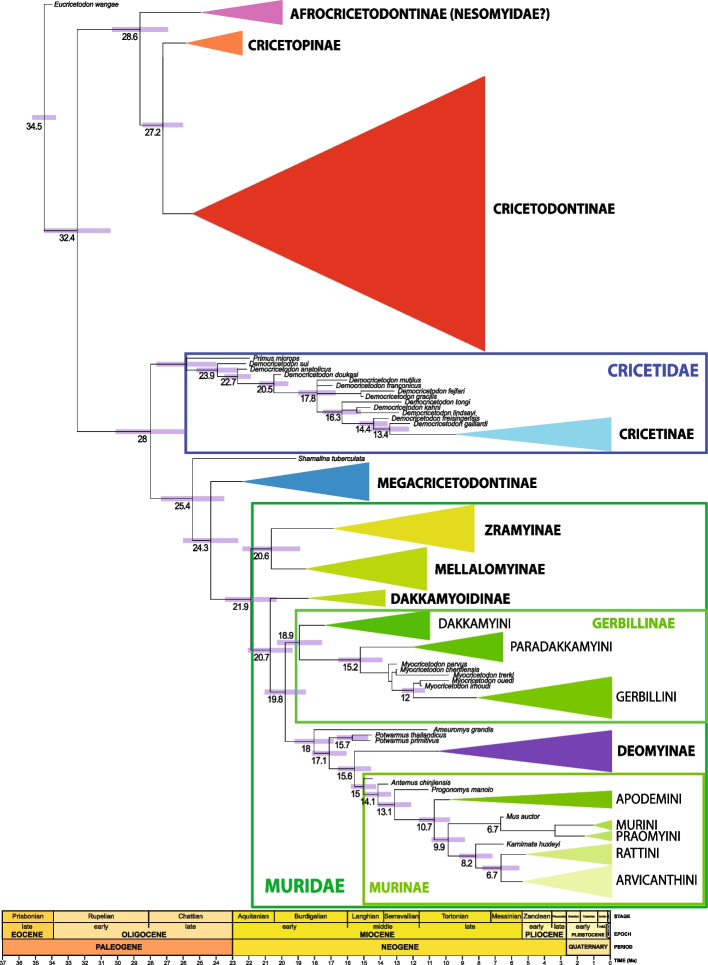
Summarized phylogeny of Muroidea. Maximum compatible consensus tree summarizing the relationships among subfamilies and tribes inside muroid rodents. Cones represent the sampled diversity of each subfamily and tribe and the depth of each cone corresponds to their most recent common ancestor

The topology of the tree shows two large sister clades. The backbone tree that summarizes the subfamily relationship is shown in Fig. 1. Within the first major clade, Cricetidae is the earliest diverging clade. This latter is the sister group of a larger clade that splits into two clades, the Megacricetodontinae on one hand and the Muridae on the other hand. The second clade includes two clades that are sister clades: †Cricetopinae (sensu Maridet and Ni [42]) and †Cricetodontinae. These results evidence that the family Cricetidae, as traditionally conceptualized by paleontologists, that is, considering all cricetid-like rodents as members of this family [42, 43], is paraphyletic and that †Megacricetodontinae and †Cricetodontinae are not part of it and should be excluded. All synapomorphies shared by these clades are listed in Additional File 1: Fig. S1.

### Cricetidae

The topology of our tree evidences that democricetodontines are, as suspected by some authors (e.g., [43, 44], closely related to extant hamsters).

**Fig. 2:**
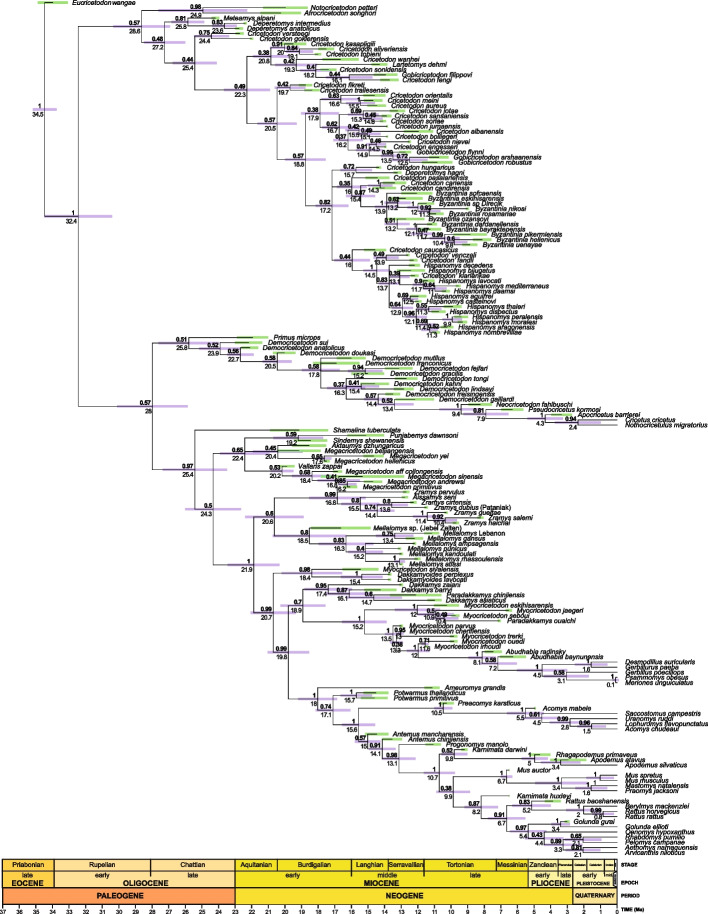
Relaxed-clock Bayesian inference analysis of the morphological dataset with tip dating using the fossilized birth–death tree model. Maximum compatible consensus tree. Numbers above nodes indicate posterior probabilities, numbers below nodes indicate median estimates for the divergence times, and node bars indicate the 95% highest posterior density for divergence times for internal nodes (magenta) and tips (green)

According to our results (Figs. 1 and 2), this subfamily originated ~ 25.8 Ma in China (Figs. 2 and 4). †*Primus microps* appears as the basalmost taxon followed by the earliest deriving †*Democricetodon*, which corresponds to †*Democricetodon sui* from the Early Miocene of the Junggar Basin, China (21.9–21.16 Ma), and represents the oldest record of †*Democricetodon* in Asia [45]. Subsequently deriving taxa include the Turkish †*D. anatolicus* and †*D. doukasi*, followed by the remaining species of †*Democricetodon*. The subsequent divergence of †*D. franconicus*, †*D. mutilus*, and then †*D. gracilis* and †*D. fejfari* (as sister species) reveals the first entrance of this subfamily of rodents in Europe circa 17.8 Ma, followed by a migration from Europe toward South and West Central Asia ~ 15.2 Ma (Fig. 2). The origin of extant cricetines, which are here represented by *Cricetus cricetus* and *Nothocricetulus migratorious*, is to be found in some European species close to †*Democricetodon*: †*D. freisingensis* and †*D. gaillardi*. The basal divergence in the crown clade would have occurred around 13.4 Ma (Fig. 1). This latter clade shares some non-exclusive synapomorphies such as the lack of lingual anteroloph (12 (0➜1)) on the M1 and central atoll on the M3 (52 (0➜2)), the presence of the posterior spur of the anterocone (anterior ectoloph, (14 (0➜1)) on the M1 or a medium length mesoloph ((21 (0➜1), 38 (0➜1)) on the M1 and M2 (Additional File 1: Fig. S1).

### †Megacricetodontinae

We find †Megacricetodontinae as the sister clade to Muridae (Fig. 1). This fact evidences that the former subfamily is closer to murid than to cricetid ancestry, which led this clade outside from the Cricetidae. This result agrees with the previous hypotheses by Flynn [44], in which †Megacricetodontinae is the outgroup to (Murinae, (Gerbillinae, Deomyinae)) and that it could be seen as an earliest deriving Muridae. According to the topology of our tree, †*Shamalina* is the most recent common ancestor of †Megacricetodontinae + Muridae, from which they diverge approximately 24.3 Ma (Fig. 2). Lindsay [46] suggested that †*Shamalina* (Early Miocene, Saudi Arabia) could have given rise to †*Megacricetodon* whereas Wessels [47] included this taxon within the †Myocricetodontinae. Within †Megacricetodontinae, there are three main clades, whose evolutionary relationships cannot be fully resolved. The first one consists of the sister species, †*Punjabemys downsi* and †*Sindemys shewanensis*, from the Early Miocene of Pakistan [47]. Both were considered by Wessels [47] as belonging to †Myocricetodontinae. However, according to our results (Fig. 2), they belong to †megacricetodontines, supporting the ideas of Lindsay [48]. The second clade is composed of a succession of Asian plesiomorphic species that led to †*Megacricetodon hellenicus* from the Early Miocene of Aliveri, Greece [49]. This lineage evidences the first entrance of †*Megacricetodon* into the Aegean–Anatolian region which is set up at ~ 20.4 Ma. The third clade is constituted by a succession of lineages, the most basal of which is †*Vallaris zappai* from the Early Miocene of Turkey, which splits into two lineages supporting two independent migration events. The first one led to †*Megacricetodon* aff. *collongensis* (~ 17.2 Ma), the earliest deriving species of the typical central European † “*Megacricetodon bavaricus* group” [50] and the second one includes the split between †*M. andrewsi* and †*M. primitivus* at ~ 16 Ma. Interestingly, Oliver and Peláez-Campomanes [51] inferred at least three migration events for early †*Megacricetodon* as the basis for key morphological differences they found between this taxon.

### Muridae

Potential candidates have been proposed for the origin of the different subfamilies and tribes inside Muridae. For instance, †*Abudhabia* has been suggested to be good candidate for being the ancestor of Gerbillinae [52], †*Antemus* for Deomyinae [53] or Murinae [52, 54], †*Karnimata* for the tribes Rattini [54] or Arvicanthini-Millardini-Otomyini [52, 55, 56], †*Progonomys* for the tribu Murini [54, 56] and for the tribes Praomyini-Murini-Apodemini [52, 55, 57], and so on.

The topology of our tree as seen above shows that Muridae is the sister group to the †Megacricetodontinae, from which it diverged at ~ 25.4 Ma (Figs. 1–2). Most of their oldest representatives belong to whole extinct lineages that, according to our results, would warrant subfamily status. So Muridae is divided into two sister main clades that diverge approximately 21.9 Ma. The first of them includes the sister subfamilies †Zramyinae and †Mellalomyinae whereas the second one, which is more speciose, shows six main lineages, the subfamilies †Dakkamyoidinae, Gerbillinae, Deomyinae, and Murinae (Fig. 1). The diversification of †Dakkamyoidinae and Gerbillinae occurred within very short time intervals from each other, < 2.5 Ma (Fig. 2) whereas that of Murinae and Deomyinae started much later (15.0 Ma and 10.5 Ma, respectively) (Fig. 1). All these clades show the posterior part of the m3 reduced phylogenetic and they share the synapomorphies of having the entoconid-hypoconid reduced (83(0➜1)), extremely reduced (83(1➜2)), included in a single cusp (83(1➜3)) or even absent (83(2➜4)) and the posterosinusid small (85(0➜1)) or absent (85(1➜2)) (Additional File 1: Fig. S1).

#### †Zramyinae

The earliest deriving split at ~ 21.9 Ma within the Muridae separates the sister subfamilies †Zramyinae and †Mellalomyinae from the remaining subfamilies of this diversified group (Figs. 1–2). The systematic placement of †*Zramys* has been for a long time questioned. This genus was first considered as belonging to the Cricetidae [58], then suspected to be a †Myocricetodontinae [59] before being formally reallocated to this group [47]. Nevertheless, the phylogenetic relationships of the taxa belonging to this genus have never been formerly analyzed. The genus †*Aissamys* was coined in an unpublished thesis [60]. It is therefore not available (*nomen ex dissertatione*). The type and only species of the genus, †*Aissamys seni* from Chouf Aïssa, is here considered as †*Zramys* sp. The topology of the tree shows that the polytypic African genus †*Zramys* forms a monophyletic group that belongs to the new subfamily †Zramyinae, whose origin can be inferred at approximately 16.8 Ma (Fig. 2). The †Zramyinae (node 186) share the exclusive synapomorphy of having lingual spur of the anterocone (11➜01) on the m1. Additional non-exclusive synapomorphies are the presence of posterior spur of the anterocone (14➜01) on the M1, having an elongated M2 (34➜01) with a posterior metaloph (39➜02) and the presence of an anterior spur on the entoconid on the m1 (92➜01) and m2 (93➜01) (the latter two, being only shared by †*Notocricetodon petteri*). Moreover, the most derived †Zramyinae share the exclusive synapomorphy of having a transversal protocone spur (23➜01) (Additional File 1: Fig. S1).

#### †Mellalomyinae

The extinct polytypic genus †*Mellalomys* forms a monophyletic group, which splits from †Zramyinae ~ 20.06 Ma (Figs. 1–2). Its earliest deriving taxon is †*Mellalomys* sp. from the Early Miocene of Jebel Zelten, Libya [61]. One node up this lineage splits to give rise to two clades (Fig. 2). The first one includes all African species of the genus whereas the other one comprises the taxa recorded out of Africa and evidences a migration event from Africa towards the Arabian Peninsula and China ~ 16.4 Ma.

#### †Dakkamyoidinae

The †Dakkamyoidinae is an extinct endemic subfamily from Pakistan that splits from more derived murids ~ 20.7 Ma (Figs. 1–2). Its earliest deriving taxon is †*Myocricetodon sivalensis* from the Middle Miocene of Pakistan [48, 62]. The inclusion of this species into the genus †*Myocricetodon* has been a matter of debate [63, 64]. According to the topology of the tree, †*Myocricetodon sivalensis* should be reallocated into the genus †*Dakkamyoides*. This species is sister species to the clade constituted by the two species of †*Dakkamyoides* (†*D. lavocati*, †*D. perplexus*).

#### Gerbillinae

According to our results, Gerbillinae diverged from Murinae + Deomyinae at ~ 19.8 Ma (Figs. 1–2). These three clades share the non-exclusive synapomorphy of lacking the lingual anteroloph on the M2 (31:02) (Additional File 1: Fig. S1). The earliest deriving clade inside the Gerbillinae corresponds to an extinct lineage that we call here tribe †Dakkamyini (Fig. 1). Its earliest most deriving taxon is †*Dakkamys zaiani* from the Middle Miocene of Beni Mellal, Morocco [59]. †*Dakkamys zaiani* is the sister species to the clade composed of the remaining known species of †*Dakkamys* and †*Paradakkamys chinjiensis*, which come from the Middle Miocene of Pakistan (Fig. 2). Our analysis suggests that the origin of this tribe predate Middle Miocene times. According to our results (Fig. 2), †*Paradakkamys chinjiensis* should be reallocated into the genus †*Dakkamys*. The †Dakkamyini is the sister clade of a speciose group from which they diverged approximately 18.9 Ma. This latter is constituted by two sister clades. The first of them that we call here †Paradakkamyini, includes all species of †*Myocricetodon* that have developed an enterostyle on the M1 and M2 and sometimes an ectostylid on the m1. Based on these characteristics, Lindsay [48] excluded these taxa from the genus †*Myocricetodon* and included them together with the genus †*Dakkamys* into the Dendromurinae. However, according to our results, all these species should be reallocated into the genus †*Paradakkamys* in the tribe †Paradakkamyini inside Gerbillinae. Except for its earliest deriving taxon † “*Myocricetodon*” *eskihisarensis*, which comes from the Middle Miocene of Turkey, the remaining species within this clade are African († “*M.” jaegeri*, † “*M.” ouaichi*, † “*M”. seboui*). The second group includes the †*Myocricetodon* species that are characterized by lacking enterostyle on the M1 and M2 and more derived gerbillines. Its earliest deriving taxon is †*Myocricetodon parvus* from Beni-Mellal, Morocco. This species is, in turn, sister to two clades. The first one is represented by †*M. trerki* and †*M. cherifiensis* whereas the second one is a larger clade that includes on one hand †*M. irhoudi* and †*M. ouedi* and on the other hand the clade †*Abudhabia radinski* plus more derived gerbils. This latter group includes the living gerbils and it is supposed to be close to the ancestry of all extant members of Gerbillinae. Our results (Figs. 1 and 2) agree with those of Jaeger [59], who suggested the polyphyly of †*Myocricetodon.* As seen above, one of the former lineages considered to be within †*Myocricetodon* is excluded from the genus †*Myocricetodon* and reallocated into the genus †*Paradakkamys*. Our results agree with those pointed out by [64], who considered all species belonging to the genus †*Paradakkamys* and †*Dakkamys* as more closely related to gerbils than to murines. Moreover, the topology of our tree evidence that both †*Dakkamys* and †*Paradakkamys* belong to Gerbillinae. The most derived Gerbillinae (†*Abudhabia radinski* plus more derived gerbils) have sister relationships with †*Myocricetodon irhoudi* as suggested previously by some authors [59, 65, 66]. The origin of the Gerbillinae crown group is inferred at ~ 4.5 Ma ((median age; 95% highest posterior density [HPD]: 3.08–6.1 Ma). Inside this group, our results show a basal split between (*Desmodillus*, *Gerbillurus*) on one hand and (*Gerbillus* (*Psammomys*, *Meriones*)) on the other hand. These results are in agreement with molecular studies, in which *Desmodillus* and *Gerbillurus* belong to the tribe Taterini (Taterini II sensu Alhajeri et al. [67]), which is in turn the sister group of a clade that includes the tribe Gerbillini (Gerbillini I sensu Alhajeri et al. [67]) with *Psammomys* and *Meriones* being closer to one another than either is to *Gerbillus*. According to our morphological clock, the split between Taterina and Gerbillina (here considered as subtribes) took place between the end of the Miocene and the beginning of the early Pliocene. These results are in line with the fossil record, from which we know that the oldest fossils attributed to modern gerbil genera date from the Early Pliocene (5.1 Ma) of Langebaanweg, South Africa [68].

The species belonging to the tribes †Paradakkamyini and Gerbillini share the synapomorphy of having an extreme degree of reduction on the m3 (76:34) with the exception of †*M. parvus*, in which the m3 is reduced but to a lesser extent (Additional File 1: Fig. S1) as it is in the representatives of the tribe †Dakkamyini. This reduction in the size of the third molars comes together with the reduction on the number of roots from two (on the m3) and three (on the M3) to one (characters 86(0➜2) and 89(0➜3)). The species included in the clade (†*Myocricetodon parvus* + more derived species) share the synapomorphy of lacking the entoconid on the m3 (84(1➜2)) (Additional File 1: Fig. S1). This synapomorphy is also present in all Deomyinae but the earliest deriving most one (†*Preacomys karsticus*).

#### Murinae and Deomyinae

Our results agree with molecular studies in recovering the monophyly of Gerbillinae, Murinae, and Deomyinae as well as in evidencing a close relationship between these clades (Fig. 1). However, the phylogenetic position of these clades relative to one another differs from what has been recovered using molecular analyses. Molecular results indicate a sister–group relationship between Murinae and the clade Deomyinae + Gerbillinae [21–23, 69]. However, the topology of our tree shows Gerbillinae as the sister clade to Murinae + Deomyinae. This is a matter of great interest because Deomyinae have been solidly placed inside murines by paleontologists on the basis of the morphology of their molars. Despite numerous attempts to morphologically demonstrate that *Acomys*, a representative of Deomyinae, did not belong to Murinae, the difficulty remained. As Jacobs and Flynn [70] pointed out, *Acomys* if not a murine, represents a very rare case of a nearly perfect convergence in complex dental features among rodents. Our results based on dental morphological data allow separating Deomyinae from Murinae but they place these subfamilies closer to one another than either is to the Gerbillinae.

According to our results (Figs. 1–2), Gerbillinae represents the sister clade of a large group, the earliest deriving taxon of which is †*Ameuromys grandis* from the Late Miocene of Egypt [71]. This species shares with some species of †*Zramys* and †*Mellalomys* the non-exclusive synapomorphy of having three roots, the anterior one of which is divided on the m2 (78(0➜1)) (Additional File 1: Fig. S1). †*Ameuromys grandis* splits into two lineages. The smaller one includes the species belonging to †*Potwarmus*, which is the sister group of murines + deomyines, from which it diverges approximately 17.1 Ma. Therefore, our results provide evidence that †*Potwarmus*, which was once considered a stem murine [56, 64] similar to †*Antemus* [72], does not belong to Murinae but to an early dead-end offshoot of the clade leading to Murinae and Deomyinae.

Murinae and Deomyinae share the exclusive synapomorphy of having on the M1 an anterocone divided with its lingual part larger than the labial one (6(2➜3)) These two clades together with *Potwarmus* share the exclusive synapomorphy of having the main cusps of the M1 forming rows (4(1➜2)) (Additional File 1: Fig. S1).

Deomyinae is a subfamily of rodents mainly endemic from Africa (except for the genus *Acomys*, which is also found in the Middle East and Crete). The origin of this clade is set up approximately 10.5 Ma. Its earliest deriving member is †*Preacomys karsticus* from the early Late Miocene (circa 10 Ma) of Harasib, Namibia [73], which is the sister species of the remaining taxa of this subfamily. Deomyinae share the exclusive synapomorphy of having the hypocone of the M3 lingual to the protocone (58(3➜4)) (Additional File 1: Fig. S1).

Interestingly, Jacobs and Flynn [70] pointed out that if *Acomys* is not a Murinae, it should have diverged from the latter prior to †*Progonomys* or †*Antemus*. Our results are in line with the thoughts of these authors and evidence a split between Deomyinae and Murinae prior to †*Progonomys* and †*Antemus* (see below).

†*Antemus* is known by two Pakistani species, †*Antemus mancharensis*, the oldest one from the late Early Miocene (*ca.* 15 Ma, [47]), and †*Antemus chinjiensis*, the youngest, (13.8–13.05 Ma [57]). The topology of our tree suggests that the most basal stem murine is †*Antemus mancharensis*, which is not unexpected given the absence of a real t1 in some M1 of this species, which show a low ridge instead ([47] p. 218). †*Antemus chinjienis* and †*Progonomys manolo* insert sequentially on the stem. Both taxa show a true t1 on all their M1 (novelty already fixed), the development of which already started in †*A. mancharensis* (without reaching fixation in this taxon). The presence of a t1 may have taken place at ~ 15.02 Ma, age at which we establish the origin of Murinae (Figs. 1 and 2). Our results show agreement with the fossil record, with the oldest murine rodent dating back approximately 15 Ma. According to the topology of our tree, †*Progonomys manolo* leads to two main clades, the first of which with †*Karnimata darwini* as the most basal taxon, leads to the tribe Apodemini, from which it splits at approximately 9.8 Ma. The second clade splits into two clades, the first of which shows as basal †*Mus auctor* and leads to the tribes Praomyini and Murini as sister groups whereas the second one has as basal most taxon †*Karnimata huxleyi* that give rise to the tribes Rattini and Arvicanthini. Interestingly, despite this work focuses on exploring deeper nodes inside Muridae, even a small representation of murines allows recovering the monophyly of this subfamily inside murids. In this way, Murinae shares the synapomorphies of having a t1 but no lingual branch of the anterio cingulum on the M2 (32(1➜3)), the presence of the anterolabial cuspid (A1) (96(0➜3)) and labial cuspids (C1 or/and C3) on the m2 (102(0➜2)). Crown Murinae, which are here represented by all murines but †*Antemus* and †*Progonomys*, are characterized by the presence of a t1 together with the loss of the lingual branch of the anterior cingulum on the M2 (32(3➜4)), by having t3 (33(2➜3)) on the M2, a large t1 on the M3 (44(2➜4)) and the entoconid and hypoconid merged in a single cusp on the m3 (83:1➜3). Our findings also support the monophyly of the tribes analyzed here: Arvicanthini, Rattini, Murini, Praomyini, and Apodemini (synapomorphies are shown in Additional File 1: Fig. S1). Moreover, our analysis identifies a closer relationship between Apodemini and the sister tribes Murini and Praomyini than either is to Arvicanthini, which is in agreement with molecular studies [23, 74] and paleontological data [56]. The basal position of *Golunda* inside the Arvicanthini, followed by the divergence of *Oenomys* has also been recovered in molecular studies [23, 74]. However, we recognize two clearly separate species inside †*Karnimata* that would give rise independently to the tribes Apodemini (†*K. darwini*) on one hand and Arvicanthini and Rattini (†*K. huxleyi*) on the other hand, whereas †*Progonomys* would be a stem murine. Nevertheless, the presence of a t1 together with the already loss of the lingual branch of the anterior cingulum on the M1 exhibited by †*Progonomys* (13(3➜4)), a synapomorphy shared by crown murines, leave still room for doubt concerning the exclusion of this genus from the crown group. The origin of Murini and Praomyni is, according to our results, related to another important fossil taxon †*Mus auctor*. According to Kimura et al. [57] and Flynn et al. [56], the origin of Murini and Arvicanthini would be found in †*Progonomys* and †*Karnimata*, respectively.

The inclusion here of a small sample of extinct and extant murine representatives does not allow to fully understand the tempo and mode of evolution of murines, which merits a study on its own. In fact, hundreds of fossils and living species of murine rodents need still to be added in a more comprehensive phylogenetic analysis (in preparation) to finally depict their evolutionary history of what is the most specious subfamily of mammals.

### †Cricetodontinae

Our results indicate that Cricetodontinae are not closely related to Cricetidae, Megacricetodontinae, and Muridae (Figs. 1 and 2), so the extinct subfamily Cricetodontinae should be excluded from all these groups. The subfamily Cricetodontinae diverged from the primitive Cricetopinae at approximately 27.24 Ma (Figs. 1 and 2). The earliest deriving clade within the Cricetodontinae (Fig. 2) is represented by two sister species †*Cricetodon versteegi* and †*C. goklerensis* from the Early Miocene of Turkey [75, 76]. This clade is the sister clade to a larger group that includes all remaining species of the subfamily. Its earliest deriving clade has two main branches. The first one includes a sequence of species of †*Cricetodon* from China giving rise to (†*C. wanhei* (†*C. sonidensis*, †*Mixocricetodon dehmi*, (†*C. fengi* and †*Gobiocricetodon filippovi*))) whereas the second branch includes the †*C. kasapligili* as giving rise to †*C. tobieni* and †*C. aliveriensis* as sister species. The split of these two early deriving clades and the clade comprising more derived species of †*Cricetodon* and the species of †*Byzantinia* and †*Hispanomys* is set up at 22.29 Ma. Inside this clade, the earliest deriving lineage is represented by the Turkish species †*C. fikreti* and †*C. trallesensis* that diverged from the main clade at approximately 20.48 Ma. This latter clade splits into two main branches. The first one comprises (†*C. orientalis* (*C. aureus*, *C. meini*)) as sister species of most of the Middle Miocene European species of †*Cricetodon*. The second main lineage comprises three lineages. The first one is represented by the Middle Miocene European †*C. hungaricus* and †*Deperetomys hagni*. The second one leads to †*Hispanomys* and includes the late Middle Miocene †*Cricetodon* from Central Europe †*Cricetodon caucasicus*, †*C. fandli*, †*C. klariankae*, and †*C. venzecli*, which should be considered as †*Hispanomys*. The third one, from which †*Byzantinia* originates, is unresolved, and contains the Middle Miocene †*Cricetodon pasalarensis*, †*Cricetodon cariensis*, and †*C. candirensis*, which would be early representatives of †*Byzantinia*. The clade that includes the most derived species of †*Cricetodon* that lead to all species of †*Hispanomys* and †*Byzantinia* share the synapomorphy (78(0➜2)) of having three roots with the posterior one divided on the m2 (except for †*B. sofcaensis*, which is two rooted and the most derived †*B. uenayae*, which is four rooted, 78(2➜3)) (Additional File 1: Fig. S1). Some additional synapomorphies consist in the presence of a long longitudinal or oblique backward paracone spur (15(1➜2, 3)) and an anterior spur of the metacone (16(0➜1)) on the M1 or having an elongated M2 (34(0➜1)) (Additional File 1: Fig. S1).

### †Cricetopinae Deperetomys/Meteamys clade

On the basis of a cladistics analysis, [42] coined the new subfamily †Cricetopinae to include the species †*Paracricetops*, †*Cricetops*, †*Deperetomys* (Early Miocene forms), †*Meteamys*, †*Selenomys*, †*Melissiodon*, †*Mirrabella*, †*Enginia*, †*Muhsinia*, and †*Aralocricetodon*. Two rodents from this analysis (†*Deperetomys* and †*Meteamys*) have been included in ours. The origin of †*Deperetomys* and †*Meteamys* has been widely discussed [43, 45, 76, 77]. Some authors considered that †*Deperetomys* and †*Meteamys* together with †*Cricetodon* and †*Eumyarion* probably derived from the early stock of cricetodontines [76]. Other authors considered them as belonging to a new subfamily, †Eumyarioninae [77]. Our results agree with those of [42] in finding †Early *Deperetomys* and †*Meteamys* as members of a subfamily outside of Cricetodontinae, which they called †Cricetopinae. Interestingly, our results indicate that †*Deperetomys hagni* belongs, in fact, to the genus †*Cricetodon*. The similarities between †*Deperetomys hagni* and some species of †*Cricetodon* have been noticed since the discovery of that species. Fahlbusch [78] named this species as †*Cricetodon sansaniensis hagni* and Mein and Freudenthal [43] coined †*Deperetomys* as a subgenus inside †*Cricetodon* based on the type species †*Cricetodon* (*Deperetomys*) *hagni*. In the same line of reasoning, the species †*Cricetodon hungaricus* was named as a subspecies of †*Deperetomys hagni*. Therefore, it is not surprisingly that our results evidence that †*Deperetomys hagni* belongs in fact to †*Cricetodon* and should be reallocated to this genus. As †*Deperetomys hagni* is the type species of the genus, it is plausible that after carrying out a comprehensive analysis of the species belonging to these ancient lineages is necessary to coin a new genus to reallocate all species of †*Deperetomys* but †*D. hagni*.

### †Notocricetodon pelteri + Afrocricetodon songhori

†*Notocricetodon* and †*Afrocricetodon* are the first fossils belonging to the Muroidea that have been recorded in Africa. They date from the early to middle Miocene and have been included in the fossil subfamily †Afrocricetodontinae by Lavocat [34], who already suspected that they could belong to Nesomyidae. Over the history, various tentative classifications of Nesomyidae, which were considered with different taxonomic rank (family or subfamily, depending on the authors), have been carried out ([79] and references therein) but the high level of dental convergence they show has made very difficult to infer their phylogenetic relationships [80]. Finally, molecular studies have put in evidence that Nesomyinae together with other African groups (Delanymyinae, Mystromyinae, Petromyscinae, Cricetomyinae, Dendromurinae) constitutes the family Nesomyidae, which is basal to the Muridae [23]. However, their relationship with some extinct taxa such as †*Democricetodon* or †*Myocricetodon* is still a matter of discussion ([81] and references therein).

Our results support the monophyly of the sister taxa †*Afrocricetodon* and †*Neocricetodon* and its basal position with regard to †Cricetodontinae, Cricetidae, and Muridae, which is in agreement with molecular studies [23]. These two taxa are an archaic African lineage that split from †Cricetopinae and †Cricetodontinae approximately 28.6 Ma.

The relationship between these fossil species with extant genera belonging to Nesomyidae are still unknown. In fact, due to the dental convergence of the Nesomyidae, *Saccostomus* branches in our analysis inside the clade Deomyinae. So, many more taxa belonging to extinct and extant members of Nesomyidae as well as additional morphological characters that complement the dental ones, particularly in that concerning the skull and muscles, need to be added in future phylogenetic analyses to finally depict the relationships between extinct and extant members of this African family of rodents.

### Divergence times

Fossil data are fundamental to molecular clock methodology, providing the key means of clock calibration. In order to use fossils as calibration points for molecular clock, we need to know the phylogenetic position of these fossils into a tree and to set up the time, in which the clades to which these fossils belong, originated, and diverged. So, one of the most difficult challenges facing any scientist working in phylogenetic inferences using molecular dataset is the phylogenetic position of the fossils in the tree. In fact, early fossils may be representatives of stem lineages rather than crown groups [23] or belong to a different group, for which they have been used as calibration points. In all these cases, the calibrations will be wrong.

**Fig. 3:**
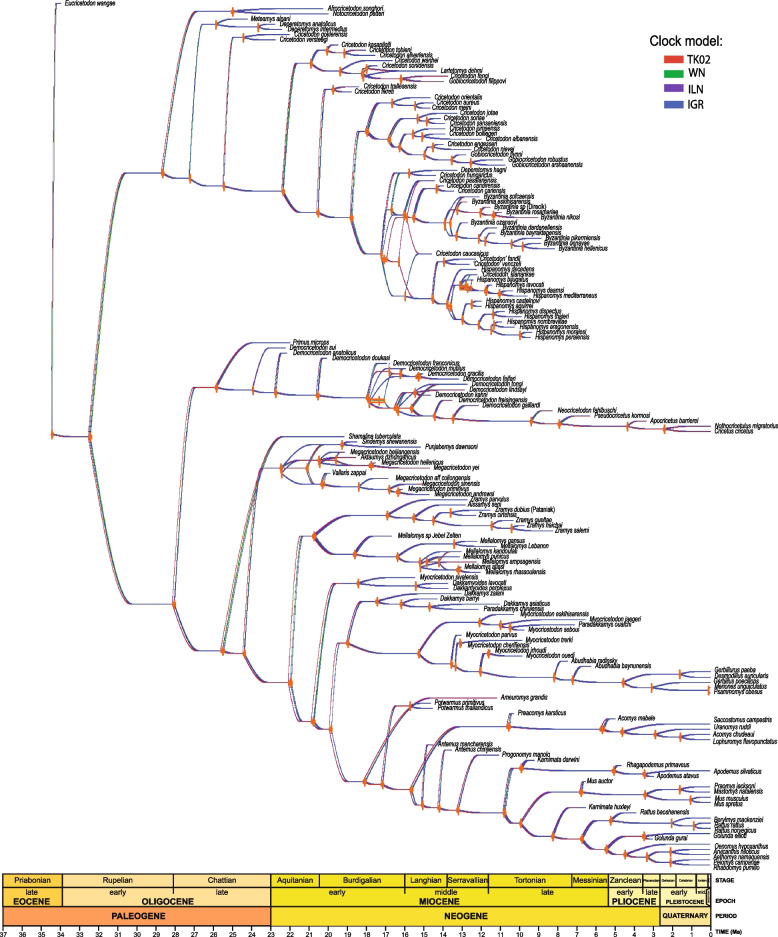
Density tree contrasting divergence times under distinct clock models. Each tree represents the maximum compatibility tree and median divergence times obtained from each model. Gray bars at nodes indicate range between maximum and minimum divergence times for each model and node values represent the midpoint between these respective maximum and minimum divergence times. IGR independent gamma rates uncorrelated clock, ILN independent lognormal relaxed clock, TK02 continuous autocorrelated clock, WN white noise uncorrelated clock

Our analyses herein infer with strong support the placement of several fossil groups never previously tested using Bayesian tip dating approaches. Importantly, we find a strong convergence of divergence time estimates for nearly all nodes within Eumuroida using multiple relaxed clock models—even when using models (i.e., WN) strongly rejected by our Bayes factors comparisons (Fig. 3, Table 1). This demonstrates that our inferred divergence times are robust to model violation and provide a precise (narrow highest posterior density intervals) timeline of muroid evolution.

**Table 1:** Results of model fitting analyses using the stepping-stone algorithm for Bayes factors. *BF* Bayes factor relative to best-fit model, *mLnL* marginal natural log-likelihood

Clock model	mLnL	BF
IGR	− 7329.76	190.34
ILN	− 7330.05	190.92
WN	− 7396.93	324.68
TK02	− 7234.59	0

We find the origin of the most recent common ancestor (MRCA) of Eumuroida (sensu [22]) at ~ 32.45 Ma (median age; 95% highest posterior density (HPD): 30.38–34.45 Ma). This implies that the origin of this large clade (inclusive of all muroids except for Platacanthomyidae and Spalacidae) predates the age inferred by molecular clock studies using only node calibrations by at least 7 Ma Schenk et al. [21] and by more than 12 Ma as estimated by Steppan and Schenk [23] and inferred 22% and 37.5% underestimate for the total age of Eumuroida, respectively.

The topology of our summary tree suggests that neither †Cricetopinae nor †Cricetodontinae are a part of Cricetidae. The split between †Cricetopinae and †Cricetodontinae took place at ~ 27.2 Ma (median age; 95% highest posterior density (HPD): 26.1, 28.5 Ma). The origin of the most recent common ancestor (MRCA) of †Cricetodontinae is at ~ 25.43 Ma (median age; 95% highest posterior density (HPD): 24.1, 26.8 Ma) and that of the †Cricetopinae is ~ 25.8 Ma. The divergence between Cricetidae and the clade Muridae + †Megacricetodontinae is at ~ 28Ma and the origin of the MRCA of Cricetidae, around 25.8 Ma. Hedges and Kumar [82], based on the supertree they built, estimated a much older age (40.2 Ma) for the origin of Cricetidae whereas [21] and [23] a much younger one (17.7 Ma and 14.6 Ma, respectively). According to these latter authors, Cricetidae would have originated after the Muridae. However, our results agree with the fossil record that evidences without any doubt that the origin of cricetids took place much earlier than that of murids.

The inclusion in our analysis of murid fossils now recognized as stem Muridae (†Zramyinae, †Mellalomyinae, †Dakkamyoidinae) evidence the existence of Muridae at least since 21.89 Ma. So, our estimate predates the age inferred by molecular clock studies given by Aghová et al. [52] (20.1 Ma), [21] (20.7 Ma), and [23] (17.4 Ma).

We find the origin of the MRCA of murines at ~ 15 Ma, one million years older to that obtained for the origin of this group by Schenk et al. [21] (14.2 Ma) and Rowe et al. [83] (14 Ma) but somewhat younger than the age estimated by Aghová et al. [52] (15.9 Ma). The divergence age between †*Progonomys* and the crown group of murines is here set up in circa 13.1 Ma and the origin of the MRCA of the crown murines that leads to all known extant species of murines in 10.7 Ma. The oldest fossil occurrences of †*Antemus* (†*A. mancharensis*), †*Progonomys* (†*P. morganae*), and †*Karnimata* (†*K. fejfari*) fall within the time ranges we have estimated by our divergence dates. If we consider †*K. darwini* as the most basal representative of Apodemini, this tribe would have originated around 9.8 Ma, which is consistent with molecular studies [74]. However, the origin of the crown group of this tribe is considerably younger (4.4–5.9 Ma). Our estimates for the timing of the origin of Praomyini and Murini lineages is 1.9–5.1 Ma and that for the origin of the tribes Rattini and Arvicanthini 5.5–7.8 Ma. Our presented divergence time estimates for extant tribes are younger than that inferred by other studies [74, 84] who inferred for instance an approximate age of 8 Ma and 8.4 Ma for the origin of Arvicanthini and circa 8 Ma and 7.6 Ma for the Praomyini. Despite the fact that fossil representatives of modern genera among murines are not reported with certainty before 5–7 Mya [53] and therefore their evolutionary time frame should not be much older than that, we remain cautious concerning the origin and divergence ages of the most derived clades inside murines until the completion of a more comprehensive analysis.

According to our results, Deomyinae originated at ~ 10.47 Ma, approximately 2 Myrs before the age estimated by Schenk et al. [21] and 2 and 3 Myrs after that estimated by Steppan and Schenk [23] and Aghová et al. [52], respectively. The earliest fossil Deomyinae showing a modern deomyine dental pattern (†*Preacomys*) has been recovered from Ethiopia and Namibia around 11–9.5 Ma [73, 85].

Gerbillinae is here considered to be the clade (†Dakkamyini (†Paradakkamyini + †Gerbillini)). The origin of its MRCA is at ~ 18.92 Ma and that of its crown group (†*Abudhabia radinski* + more derived gerbils) at ~ 8.13 Ma. Previous molecular clock hypotheses have placed the origin of the Gerbillinae either earlier, around 23.7–23.6 Ma ([86] p. SA1, 56) or later, approximately, 10.2 Ma [23]. One of the possible reasons for such disparity could be the inherent problems due to the use of the origin of clades as age constraints. A usual error is to neglect the extinct lineages belonging to these clades and, therefore, considering them younger than what they probably are. For instance, according to the topology of our tree, we could have considered Gerbillinae as the clade including †*Abudhabia radinski* plus more derived gerbils (origin at ~ 8.13 Ma), or †*Myocricetodon parvus* + more derived gerbils (origin at ~ 13.29 Ma), or (†Paradakkamyini + (†*Myocricetodon parvus* + more derived gerbils)) (origin ~ 15.21 Ma) or (†Dakkamyini + (†Paradakkamyini + (†*Myocricetodon parvus* + more derived gerbils))), whose origin is at ~ 18.92 Ma.

Each of these clades has different origin-time estimates and the results of molecular clock analyses will be impacted depending on which one is taken as calibration point. The accurate choice of these calibration points requires deep paleontological knowledge.

### Historical biogeography

**Fig. 4:**
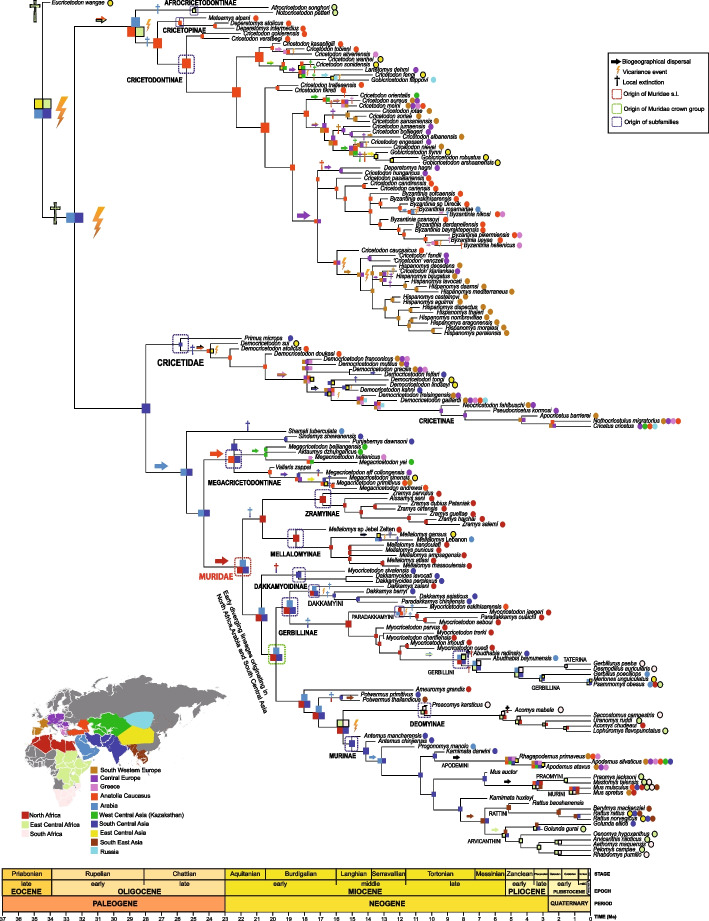
Historical biogeography of Muroidea. The schematic map shows the 13 biogeographical areas for the DECX analysis. Colored areas on the map correspond to colored squares for each node, representing inferred ancestral range(s) with the DECX model. Colored circles at tips correspond to present-day distributions

Fossil data are vital to infer the biogeographical history of a given group [87]. The results of the dispersal-extinction-*eXtended* cladogenesis (DECX) analysis suggest that the origin of the extinct subfamilies †Cricetopinae and †Cricetodontinae is estimated in Anatolia, whereas that of Cricetidae, †Megacricetodontinae, and Muridae includes several regions (Fig. 4).

#### †Cricetodontinae

Our results show some inter and intra-continental exchanges since their origin in Anatolia some 25.5 Ma. The first dispersal from Anatolia towards Central Asia occurred circa 19.32Ma. This event could have promoted the diversification of Chinese plesiomorphic species of † “*Cricetodon*”. The first migration of †*Cricetodon* from Turkey towards Greece took place in the early Miocene (*ca.* 19.3 Ma). Most European cricetodontines come from an independent Anatolian lineage that dispersed into the central and southwestern part of the continent between 16.74 and 17.89 Ma. Another dispersal event towards western China is inferred between 14.93 Ma and 13.5 Ma. A subsequent dispersal event of †*Cricetodon* from Anatolia to Central and SW Europe (*ca.* 15.96 Ma) would have led to the origin of the genus †*Hispanomys* but continued evolving in Anatolia as the genus †*Byzantinia*, whose origin has been estimated approximately 15.97 Ma. According to our results, the Late Miocene cricetodontines are not closely related to the Middle Miocene European ones. In fact, they evolved from an Anatolian ancestor that dispersed in Southwestern Europe between 17.89 and 16.74 Ma.

#### Cricetidae

According to our results, extant cricetines evolved from some lineages belonging to Democricetodontinae as already suspected [88]. So, we here consider democricetodontines as Cricetidae sensu stricto and inferred their ancestral origins to be in South Central Asia (Fig. 3) from where they rapidly dispersed into West Central Asia and Anatolia. The DECX analysis indicates a dynamic biogeographical history for cricetids with speciation mainly occurring by dispersal. The first entrance of these rodents from Anatolia into Greece and Central and Southwestern Europe is estimated between 19 and 16.7 Ma. The delayed entrance of this group into Europe could have been the result of the geographical isolation of the Dinarian-Anatolian Island [89]. Later on, these rodents dispersed eastwards from Europe towards South and East Central Asia (17.58–15.28 Ma) but continued evolving in Europe from where extant cricetines are supposed to have originated and then dispersed into Asia and the Middle East.

However, as our sample only includes a limited sample of extinct and extant cricetids, the biogeographical inferences concerning this group should be taken with caution.

#### †Megacricetodontinae

The ancestral area of this subfamily, which is recovered as the sister group of Muridae, comprises a range including the regions of Anatolia and South Central Asia. Its biogeographical history is characterized by no vicariance but some dispersal events. Early Miocene dispersals took place from South Central Asia to West Central Asia (22.4–20.4 Ma) and from there to Greece (20.4 and 17.64 Ma). The first entrance of the genus †*Megacricetodon* into Greece is estimated between 18.17 and 17.31 Ma. Two additional dispersal events in Europe for early species of †*Megacricetodon* have been identified: the first from Asia to Central Europe (20.18–18.37 Ma) and the second from Anatolia to Southwestern Europe (*ca.* 16.2 Ma). These results are in agreement with a recent study [51], who inferred at least three migration events in Europe for early †*Megacricetodon* forms. As with cricetids, our study only includes a partial representation of megacricetodontines and the results should be interpreted with caution as well.

#### Muridae

The most likely ancestral geographic origin at the root of Muridae covers South Central Asia, Arabia, and North Africa (*ca.* 21.9 Ma). Our results also point to a dynamic biogeographical history, in which vicariance events (*n* = 4) were less numerous than dispersal (*n* = 11). The fact that North Africa may have been an important ancestral area for the historical biogeography of Muridae was expected. In fact, after the collision of the Afro-Arabian plate with Eurasia [90], the subsequent closure of the Tethys Sea and the emergence of a land bridge between these continents have facilitated dispersals from Asia towards Africa and vice versa. Even though the fossil record brings to light the beginning of a foremost faunal interchange *ca.* 20–19 Ma, there is evidence that indicates intermittent trans-Thetyan dispersals before this time ([91] and references therein). So, it is plausible that ancestral murids had already spread from Asia and Arabia into North Africa shortly before 22 Ma. The analysis of the three main subfamilies within Muridae (Gerbillinae, Murinae, and Deomyinae) together with the three extinct murid subfamilies (†Zramyinae, †Mellalomyinae, †Dakkamyoidinae), evidence that some subfamilies such as the †Mellalomyinae (18.5 Ma), †Zramyinae (16.8 Ma), Gerbillinae (18.9 Ma), and Deomyinae (10.5 Ma) mostly or completely diversified into Africa. In fact, early murid clades are of African origin. So, †Zramyinae are endemic from Africa where they originated some 16.85 Ma and then diversified. The ancestral origin of the †Mellalomyinae (18.53 Ma) is also estimated in Africa. However, this extinct lineage seems to have dispersed into Arabia and South Asia between this time and 2 My after and later dispersed into East Asia (16.3–13.4 Ma) where †*Mellalomys gansus* has been found. The ancestral area of the †Dakkamyoidinae is recovered in South Central Asia where they evolved and diversified before become extinct in this region. The most likely ancestral geographic origin for the crown group of Muridae is similar with that evidenced for the root of all murids.

The origin of Gerbillinae, which includes the two extinct tribes †Dakkamyini and †Paradakkamyini as well as the tribe Gerbillini, is also inferred in South Central Asia, Arabia, and North Africa as all early-diverged murids and the tribe †Dakkamyini. DECX analysis points out to a North African origin for all gerbillines, except for †Dakkamyini that had a broader ancestral range, from where they dispersed to Anatolia (15.2–12.0 Ma), and to Arabia and South Central Asia (12.0–8.13 Ma) to give rise to the Gerbillini.

The analysis recovers an ancestral area for †*Potwarmus* that includes South Central Asia, Arabia, and North Africa. The oldest record of this taxon comes from Pakistan (locality 592) from where it is known since 16 Ma [56]. †*Potwarmus* dispersed eastwards about 15.7 Ma and enter into South Central Asia where it has been recorded in Thailand [92]. At the same time, this taxon dispersed westwards through Arabia entering into North Africa. In fact, remains of †*Potwarmus* have been found in early Middle Miocene deposits et al.-Jadidah, Arabia (12-14 Ma; [64]) and Jebel Zelten, Libya (14–15 Ma; [61]).

Deomyinae, mostly endemic from Africa, are supposed to have lived and diversified in this continent. A dispersal event from North Africa towards its South and East parts took place between 15.57and 10.47 Ma.

Murinae, also known as “true murids” originated in South Central Asia *ca.* 15.6 Ma. The oldest known murine rodents have been recorded in the Potwar Plateau (Pakistan) where they had a remarkable diversity through time [56]. Primitive murine rodents seem to have endemically evolved at least during 2 Ma in the Indian subcontinent, which was rather isolated by high mountains to the north, large rivers on the east, and desertic areas on the west [56]. The exodus of †*Progonomys* out of South Central Asia could have been bolstered by the sea level drop that took place some 11.6 Ma [93]. †*Progonomys*, dispersed out of Pakistan between 13.1 Ma and 10.5 Ma, date in which this taxon is already recorded in Turkey and Lebanon [94]. In fact, the arrival of †*Progonomys* into the Arabian Peninsula is possibly related with the final closure of the eastern Tethys and the end of the marine connection between the Mediterranean Sea and the Indian Ocean along northern Arabia [95] and see discussion in López-Antoñanzas et al. [94].

Our results indicate an important wave of dispersal for Muridae that seems to have been at the origin and diversification of all important subfamilies. In fact, early Miocene ancestors of murids entered in Africa, colonizing a new environment. Interestingly, all early diverging lineages that gave rise to the main murid clades share a common biogeographical range that covers North Africa, Arabia, and South Central Asia. So, the collision between Afro-Arabia and Eurasia likely played an important role in the origin and diversification of murids, the largest family of mammals.

During the Miocene (23.03–5.3 Ma), plate tectonics drove sea level changes and orogeny, all of which significantly altered the climate and marked the start of the late Cenozoic cold house mode and the establishment of modern terrestrial biomes [96–98]. Until the beginning of the mid-Miocene Climatic Optimum (17–15 Ma), eustatic sea-level changes indicate the presence of a moderate to large ice sheet in Antarctica [99]. The existing land bridge between Africa and Eurasia together with some periods of sea-level drop could have favored the entrance of the murid ancestors from South Asia into North Africa through the Levant. During the mid-Miocene Climatic Transition (~ 15–13 Ma), three events of cooling and sea-level fall have also been recorded at 14.8 (Mi2a), 13.8 (Mi3), and 12.8 Ma (Mi4) that culminated with the establishment of the permanent East Antarctic ice sheet [99]. These sea-level low-stand periods could have opened terrestrial routes that could have allowed the migration of the ancestors of †*Hispanomys* from Anatolia into Europe (Mi2a) and the eastwards dispersals (approximately 14–13.5 Ma) of some species of †*Mellalomys*, †*Democricetodon*, and † “*Cricetodon*” into East Central Asia (Mi3). These dispersals could have been triggered by the well-documented [99] contraction of the tropics in East Asia due to an increase in monsoon seasonality, coincident with the establishment of a quasi-permanent East Antartic ice sheet [99]. The entrance of the ancestors of Gerbillini from North Africa to Arabia and from there to South Central Asia (*c.* 8.1 Ma) could have also been triggered by the global drying and cooling climate occurring at this time [97–100]. Likewise, the origin and diversification of the African subfamily Deomyinae is linked to a dispersal event from South Central Asia that took place in the Late Miocene *ca.* 10.5 Ma. The exodus of the ancestor of this subfamily out of Asia could have been favored by the Serravallian-Tortonian sea level fall that has been documented worldwide [101].

## Conclusions

Our results evidence that neither †Cricetopinae nor †Cricetodontinae can be considered as belonging to the Cricetidae, the latter being related to Democricetodontinae. The split between these two extinct clades took place at approximately 27.2 Ma. In this work, we provide unprecedented agreement between morphological and molecular data concerning the monophyly of the Deomyinae and Gerbillinae as distinct clades within Murinae. For the first time, relaxed clock approaches using tip dating (which can account for uncertainty in the placement of fossil taxa) were used to estimate divergence times for the major groups of muroids. Even under strong violations of the best-fitting clock model, we find low levels of uncertainty for divergence times, thus providing an accurate timeline for muroid evolution. The origin of the Muridae (~ 21.9 Ma) is estimated with greater precision thanks to the inclusion of fossils now recognized as stem Muridae (†Zramyinae, †Mellalomyinae, †Dakkamyoidinae). Early diverging murid lineages originated during the Early Miocene in low latitudes (North Africa, Arabia, and South Central Asia). The ancestor of murids dispersed from South Central Asia to North Africa before 21.9 Ma. The collision between these two continents seems to have played an important role in the origin and diversification of this family of rodents. The origin of the clade that will lead to all extant murids is estimated at ~ 19.8 Ma and that of murines at ~ 15 Ma. Our results show a dynamic biogeographical history for Muroidea, in which dispersal events seem to have been more numerous than vicariance. Eastwards early Middle Miocene long-distance dispersals towards East Asia could have been favored by the increase in seasonality during the Middle Miocene Climatic Transition (MMCT) that resulted in a contraction of the tropics in this part of the continent.

## Methods

### Material

The systematic study presented below is based on the examination of original specimens and casts and data from the literature listed in Additional file 1: Table S1. First, second, and third lower molars are designated as m1, m2, and m3, respectively, and first, second, and third upper molars as M1, M2, and M3, respectively.

### Morphological dataset

The dataset presented here represents a major expansion of the morphological data matrix for cricetodontines [16]. The inclusion of Muridae has required the addition of new characters and state of characters. It has been expanded from 77 to 166 taxa and from 82 to 108 morphological characters, thus representing an increase to more than double in taxonomic sampling and coverage across all major lineages of Muroidea. The data matrix (Additional file 1: File S1) has been built using Mesquite 3.0 [102] and the morphological characters are listed in it.

### Character evolution model

We used the Markov model Mk(v) of character evolution [103] for morphological characters, implementing assortment bias correction for the inclusion of variable characters only. We allowed for among character rate variation by using a gamma distribution with four rate categories and with shape (alpha) sampled from an exponential distribution with mean = 1.0.

### Clock models

We assessed the fit of various clock models to our data, including a new suit of clock models available in the developer’s version of MrBayes (future MrBayes 3.2.8 release) [10] compiled from source code available at (https://github.com/NBISweden/MrBayes). These models include the continuous autocorrelated clock model (TK02) [104] and three uncorrelated clock models: the white noise model (former IGR model in previous versions of MrBayes) [105] and the new independent gamma rate (IGR) and independent lognormal clock (ILN) models [106] (Additional File 2: files S1-S8). These different models represent radically different interpretations of how and where in the tree evolution rates are allowed to change across lineages, which can substantially impact divergence time estimates using morphological and/or molecular data [13, 106].

We tested model fit using the stepping-stone sampling strategy to assess the marginal model likelihoods [107] and calculated Bayes Factors (BF)—30 steps (+ 5 as burn in) between for 250 million generations. Using standard significance thresholds [108], we found a strong support (BF > 5) for the autocorrelated clock model (TK02) relative to the IGR and ILN models (BFs ≈ 8) and a very strong preference for the TK02 model relative to the WN model (BF = 44.8) (Table 1).

The mean and standard deviation for the probability distribution defining the prior on the clock rate were given informative values based on previous non-clock analysis, following Ronquist et al. [109] and subsequent studies [11, 13, 110]—the median value for tree height in substitutions from posterior trees divided by the age of the tree based on the median of the distribution for the root prior (16.6429/37.6 = 0.4426). The mean of the lognormal distribution was given the value based on the non-clock tree estimate in natural log scale: ln(0.4426) =  − 0.8150. Finally, we chose a broad standard deviation around the mean (*σ*. = 1.5).

### Tree model and age calibrations

The age of the root has been set with a soft lower bound. The minimum age of the root corresponds to the oldest age for the oldest fossil belonging to *Eucricetodon* (34 Ma) and the maximum root has been estimated at 41.2 Ma (Middle/Late Eocene boundary), which is the maximum soft age for the Eucricetodontinae clade. We used an offset exponential distribution for the prior on-root age, which gives a higher sampling probability for values closer to the minimum age and a relatively low (but non-zero) probability of age values higher than 41.2 Ma.

All fossil calibrations (apart from the root node) were based on tip dates only, which account for the uncertainty in the placement of fossil taxa and avoids the issue of assigning maximum age constraints that are necessary for node-based age calibrations [109]. Additionally, we avoided recently detected strong biases that can be introduced by point age calibrations on the age of the fossils [111] by using a uniform prior distribution on the age range of the stratigraphic occurrence of the oldest fossils for each species (Additional File 2). We implemented the FBD process for the tree model with a flat prior (uniform distribution: samples ∈ U[0, 1]) on the parameters of relative extinction (= turnover) and probability of sampling fossils, whereas the net diversification rate was sampled from an exponential distribution with mean = 1.0.

Further, we performed a dense taxonomic sampling of closely related fossil lineages within a relatively short geological time span, which meets the conditions in which inferring direct ancestor-descendent relationships should be expected, and so we modeled our tree prior accordingly by allowing fossils to be either fully extinct tips or sampled ancestors [112, 113].

### MCMC and diagnostic parameters

All analyses were conducted using the developer’s version of MrBayes [10] available at (https://github.com/NBISweden/MrBayes) using the Della computer cluster at Princeton University. Convergence of independent runs was assessed using: average standard deviation of split frequencies (ASDSF ≈ 0.01), potential scale reduction factors (PSRF ≈ 1 for all parameters), and effective sample size (ESS) for each parameter greater than 200 and analyzed using Tracer 1.7.1 [114] and the R package RWTY (R We There Yet) [115] (see Additional File 3: Fig. S1).

### Stochastic character mapping

Stochastic character mapping was performed using the Stochastic character mapping of discrete traits on phylogenies (SIMMAP) approach [116] as implemented in the *make.simmap* function of the R package phytools 2.1.1 [117]. The R script is provided in Additional File 3:File S1. For each character, three different models were tested, concerning the rate of changes between states, estimated by Bayesian Markov chain Monte Carlo (MCMC): equal-rates model (ER, in which all rates are equal), symmetrical model (SYM where rates for symmetrical transition are the same), or the all-rates-different model (ARD, in which each rate can take on a potentially different value) [118]. The best-fit model was then chosen based on the Akaike information criterion and used for the stochastic character mapping. The resulting posterior probabilities of each state are represented at the nodes of the trees. Most the synapomorphies mentioned in the text does not show a large amount of uncertainty in their probability of having a given character state. However, for the few ones, which show a fair amount of uncertainty, we have taken a threshold value of 50%, above which, we consider that the node has a given character state. For some characters (marked as ER), symmetrical rate changes were favored but resulted in misbehaving analyses. In these cases, the equal rates option was chosen instead (Additional File 3: Table S1).

### Inference of historical biogeography

We estimated the ancestral ranges of origin and geographic range evolution for muroids using the maximum likelihood (ML) approach of dispersal-extinction-cladogenesis model (DEC [119]) as implemented in the *DEC eXtended* model (DECX [120]). DECX can infer the ancestral ranges using the geographic ranges of the sampled species taking into account non-ultrametric trees that include fossils as species, by modeling anagenetic events, classical vicariance, and cladogenetic events [119]. The most likely ancestral states at each node and the geographic range evolution of Muroidea have been estimated using a time-calibrated tree, a species distribution matrix, and connectivity matrices, representing the palaeogeographic history. As the input data, we have used our resulting time-calibrated Bayesian majority-rule consensus tree, the distribution of each species for a set of geographic areas, and a geographic model that is represented by connectivity and dispersal scalar matrices spanning the evolutionary history of the group. The geographic distribution of the muroid species included in our study has been categorized by coding the presence or the absence of each species in 13 areas as follows: (1) South Western Europe (SWE); (2) Central Europe (CE); (3) Greece (GR); (4) Anatolia Caucasus (AC); (5) West Central Asia (WCA); (6) Arabia (AR); (7) South Central Asia (SCA); (8) East Central Asia (ECA); (9) North Africa (NA); (10) East Africa (EA); (11) South Africa (SA); (12) Russia (RU); and (13) South East Asia (SEA). The geographic model has been built using connectivity matrices [118] that take into account paleogeography and the possibility for a species to colonize a new area, with disconnection between areas coded as 0 and connection between areas coded as 1 (Additional File 3: Table S2).

## Supplementary Information

Additional file 1: Figure S1, stochastic mapping of all synapomorphies. Pie charts at nodes indicate the relative Bayesian posterior probability of each character state. Duplicated characters are those for which the mapping with the equal rates (ER) model was added as well. Characters missing are those that presented some non-applicable states, for which the analysis did not work. File S1, morphological data matrix in Nexus format used in this study. File S2, list of morphological characters used in the analyses. Table S1, table with full taxonomic sampling, including specimen accession numbers and ages.

Additional 2: Files S1-S8. All input (S1-S4) and output (S5-S8) files from relaxed clock Bayesian inference analyses, including all necessary code to reproduce the results.

Additional file 3: Figure S2, convergence diagnostics from Bayesian inferences using the R package RWTY [115]. Script S1, R Script used in this work to carry out the stochastic character mapping performed using the SIMMAP approach [116] as implemented in the make.simmap function of the R package phytools 2.1.1 [117]. Table S2, table showing the characters for which the equal rate model has been chosen despite not being the best fitting model. Table S3, geographic distribution of the muroid species included in this study and categorized by coding the presence or the absence of each species in 13 areas. The geographic model has been built using connectivity matrices [118]. 

## Data Availability

The authors confirm that the data supporting the findings of this study are available within the article and its Additional Files.

## Abbreviations

ARD: All-rates-different model
ASDSF: Average standard deviation of split frequencies
BF: Bayes factors
DEC: Dispersal-extinction-cladogenesis
DECX: Dispersal-extinction-*eXtended* cladogenesis
ESS: Effective sample size
ER: Equal-rates model
IGR: New independent gamma rate uncorrelated clock model
ILN: Independent lognormal uncorrelated clock model
m1, m2, m3: First, second, and third lower molars
M1, M2, M3: First, second, and third upper molars
MCMC: Markov chain Monte Carlo
MK (v): Markov model that conditions on variable characters
ML: Maximum likelihood
MMCT: Middle Miocene climatic transition
MRCA: Most recent common ancestor
PSRF: Potential scale reduction factors
RWTY: R We There Yet
SIMMAP: Stochastic character mapping of discrete traits on phylogenies
SYM: Symmetrical model
TK02: Continuous autocorrelated clock model
WN: White noise uncorrelated clock model
†: Whole extinct clade
